# Determination of a safe sedative combination of dexmedetomidine, ketamine and butorphanol for minor procedures in dogs by use of a stepwise optimization method

**DOI:** 10.1186/s13028-023-00697-8

**Published:** 2023-09-22

**Authors:** Tobias Jonas Imboden, William Robert Pownall, Stéphanie Rubin, Claudia Spadavecchia, Bernhard Schöllhorn, Helene Rohrbach

**Affiliations:** 1 Border Veterinary Service, Postfach, Fracht West, Zurich Airport, 8058 Switzerland; 2 Surgery Department, Department of Veterinary Sciences, Vetsuisse Faculty, Small Animal Clinic, University of Bern, Laenggassstrasse 128, Bern, 3012 Switzerland; 3 Engelgiessstrasse 17, Scharnachtal, 3722 Switzerland; 4 Anaesthesiology Section, Department of Veterinary Sciences, Vetsuisse Faculty, University of Bern, Laenggassstrasse 124, Bern, 3012 Switzerland; 5 Vet Zentrum Berchtesgadener Land, Weitwiesenring 4, 83435 Bad Reichenhall, Deutschland

**Keywords:** Optimization method, Sedation, Safety

## Abstract

**Background:**

In veterinary practice, most minor procedures such as radiographs, skin biopsies, and wound treatments require sedation. The combination of butorphanol, ketamine, and dexmedetomidine is commonly used, but the ideal dosages for this combination have not been defined. This randomized prospective clinical 3-phases trial initially tested eight clinically relevant combinations of intramuscular administration in 50 dogs (phase 1). The quality of each combination was rated using a purposefully developed negative score (NS; 0-21.5, the lower the NS the better the quality of sedation) to judge the quality of sedation, the occurrence of side effects, and the need for additional anaesthetics. Based on the results of the NS, the eight combinations were divided into “promising” and “unsatisfactory” subgroups. In phase 2, a new combination (N) was calculated and tested in six dogs replacing the worst of the eight initial combinations. This procedure was repeated until the NS could not be improved any further. In phase 3, the best combination was tested in 100 adult dogs undergoing diagnostic or therapeutic procedures.

**Results:**

The optimal combination established was dexmedetomidine 0.005 mg/kg, ketamine 1 mg/kg, and butorphanol 0.3 mg/kg with a median NS of 1.5 (interquartile range 1.5–2.4). In all 112 dogs receiving this combination, the quality of sedation was satisfactory and no severe side effects were detected.

**Conclusions:**

The application of this optimization method allowed the calculation of an optimal drug combination to sedate cardiovascularly healthy dogs. After having being tested in 112 animals, this combination can consequently be considered safe. Therefore, this combination can now be used in daily clinical practice for cardiovascularly healthy adult dogs undergoing minor procedures.

**Supplementary Information:**

The online version contains supplementary material available at 10.1186/s13028-023-00697-8.

## Background

In daily veterinary practice, minor procedures such as radiographs, skin biopsies, and wound treatments are regularly performed in dogs. To facilitate handling as well as to reduce animal stress and pain, sedative and analgesic drugs are often administered. The combination of two or more drugs can improve the quality of sedation through their synergistic effects; also, a reduced risk of side effects due to lower individual doses can be expected [1–5].

Optimization methods aiming to identify ideal dosages for a given number of drugs as well as to obtain the optimal combination without testing too many different dosages have been developed and improved over time [6–12]. Accordingly, a stepwise optimization method was used to improve post-operative pain therapy in humans [13]. In that study, eight dose-combinations including morphine and ketamine were chosen based on clinical experience. The patients received one of the eight dose-combinations before they were asked to rate the intensity of pain by use of a visual analogue scale (VAS). According to these scores, the combinations were divided into “good” and “bad” subgroups. A new combination was then calculated based on the previously defined subgroups, replacing the worst combination. Likewise, new combinations were determined and tested until pain scores would not improve with further changes [13]. This method has been adapted for the evaluation of an optimized sedation protocol in feline clinical patients by use of a combination of alfaxalone, butorphanol, and dexmedetomidine [12].

In companion animal practice, alpha-2 adrenoreceptor agonists are commonly used sedative drugs [14]. Dexmedetomidine was the last alpha-2 agonist released to the European veterinary market in 2002. It has been described to be the sedative compound of the racemic mixture medetomidine, leading to shorter duration of sedation and to less negative effects on the cardiovascular system than medetomidine itself [15–18].

The combination of alpha-2 agonists and ketamine leads to a more reliable, steady and profound sedation than alpha-2 agonists alone [14, 19–21]. Ketamine is a dissociative anaesthetic agent able to provoke a dose-dependent loss of consciousness while maintaining cranial nerve reflexes such as the swallowing reflex [22]. Due to its sympathomimetic action, tachycardia and hypertension can occur following administration. Bradycardia is a physiological consequence of alpha-2 agonist induced peripheral vasoconstriction. Therefore, ketamine can partly counteract the medetomidine-related bradycardia, but the administration of a sympathomimetic drug might lead to transient hypertension [20, 23–25].

Butorphanol is a weak opioid being commonly used in veterinary medicine due to its moderate analgesic effects and its low potential for side effects [26, 27]. With the addition of an opioid to the dexmedetomidine-ketamine combination, the sedative and analgesic effects of this protocol can be further improved [23, 28, 29]. A combination of the three drugs dexmedetomidine, ketamine, and butorphanol is regularly administered to dogs in clinical practice [30, 31]. However, for intramuscular administration, optimal dosages for best anaesthetic conditions and for minimal occurrence of side effects upon have not yet been investigated.

We hypothesized that the optimal combination of the three drugs dexmedetomidine, ketamine, and butorphanol administered intramuscularly would result in adequate depth of sedation, allowing for minor therapeutic and diagnostic procedures with minimal side effects and minimal need for additional anaesthetic drugs in healthy dogs. Therefore, using the optimization method described by Sveticic et al. [13], the aim of this study was to find optimal dosages of dexmedetomidine and ketamine in combination with butorphanol 0.3 mg/kg in terms of quality of sedation, side effects, and need for additional anaesthetics.

## Methods

The study was approved by the Committee for Animal Experimentation, Berne, Switzerland (Approval Number BE33/11) and designed as a randomized prospective clinical trial.

One hundred and eighty client-owned, adult dogs with a mean age of 4.4 years (SD ± 3.0) and a mean weight of 31.1 kg (SD ± 15.6) were included in this study (Table 1). The reasons for sedation included radiographs, computer tomographic examinations, skin biopsies, wound treatments, external fixator removals, and other diagnostic or therapeutic procedures. All dogs were fasted overnight and considered to be cardiovascularly healthy based on physical examination and thus fulfilled the criteria of the American Society of Anaesthesiologists grade I or II. Four dogs were sedated twice, all within a minimal interval of two weeks and not using the same combination of dosages. Written owner’s consent was obtained for all dogs.

**Table 1:** Weight distribution of all dogs participating in the study

Weight group	Animal numbers per weight group	Animal numbers per breed	Breeds
1–5 kg	8	4 4	Chihuahua Various breeds
5.1–10 kg	9	9	Various breeds
10.1–20 kg	21	3 2 2 2 12	Schwyzer Laufhund Cocker Spaniel Epagneul Breton Labrador Retriever Various breeds
20.1–30 kg	47	13 7 4 3 2 2 2 24	Mix breed Labrador Retriever German Shepherd English Bulldog Boxer Dalmatian dog Entlebucher Sennenhund Various breeds
30.1–40 kg	48	9 7 7 4 4 3 2 12	Malinois Mix breeds Shepherd breeds Labrador Retriever Golden Retriever German Shepherd English Bulldog Various breeds
40.1–50 kg	24	5 4 3	Schweizer Sennenhund Mix breeds Bernese Mountain dog
		2 2 2	Labrador Retriever Hovawart Shepherd breeds
50.1–90 kg	10	2 2 6	Great Dane (2) St Bernard (2) Various breeds (6)

All 180 dogs participating in this study have been categorized by weight and breed. Breeds only represented once were summarized as “various breeds”

The three drugs dexmedetomidine (Dexdomitor, Orion Pharma, Finland), ketamine (Ketasol-100, Dr. E. Graeub, Bern, Switzerland) and butorphanol (Morphasol-10, Dr. E. Graeub, Bern, Switzerland) were combined. The clinically used dosages of dexmedetomidine (0.002–0.010 mg/kg) and ketamine (0–6 mg/kg) varied, but butorphanol was kept constant at 0.3 mg/kg (Table 2). The drugs were drawn up separately before being mixed in one syringe immediately prior to intramuscular administration. The experiments were separated into three phases: In phase 1, eight different combinations (A-H) were tested. The person performing the procedure was unaware of the combination he or she was administering. Each combination was tested in six dogs. Erroneously, the combination C was tested in eight dogs instead of six. A total of 50 dogs underwent sedation in this phase. In phase 2, new combinations (I-L_2_) were calculated and tested. Due to the fact that the same investigator (TI) performed the calculations and administered the treatments, blinding was not possible during this period. The calculations were continued until no further improvement could be achieved. A total of 30 dogs were treated in this phase. Finally, in phase 3, the best combination (L, L_2_) previously evaluated in phase 2 in 12 dogs, was tested in 100 healthy adult dogs undergoing minor procedures (combination L_3_).

**Table 2:** Overview of all combinations

Combination	dexmedetomidine, mg/kg	ketamine, mg/kg	butorphanol, mg/kg
A	0.002	4	0.3
B	0.004	3	0.3
C	0.006	2	0.3
D	0.008	1	0.3
E	0.010	5	0.3
F	0.004	6	0.3
G	0.008	3	0.3
H	0.010	2	0.3
J	0.005	0	0.3
K	0.006	1	0.3
L’	0.004	0	0.3
L	0.005	1	0.3
M’	0.002	0	0.3
M	0.004	1	0.3
L_2_	0.005	1	0.3
L_3_	0.005	1	0.3

The empirically chosen combinations A-H were analyzed in phase 1. Combinations J-M were calculated during the stepwise optimization procedure. Combinations L’ and M’ were rejected without testing because the ketamine dosage was zero and the dexmedetomidine dosage was lower than in combination J, which had been tested but rejected due to insufficient sedation. L_3_ was tested in 100 animals without comparison to other combinations

The drug combination was administered to the triceps muscle in all dogs. They were then left in a quiet environment without their owners while the onset of sedation was observed and while all of their reactions were documented. Ten minutes after injection, a catheter of appropriate size (18-22G) was placed into a cephalic or saphenous vein. In case of major resistance, manipulations were postponed for five minutes to allow for proper onset of sedation. According to the protocol, an adjunctive dose of 50% of the initial combination could be administered IM if the animal did not tolerate catheter placement. Twenty minutes after drug injection, the dogs were positioned for the procedure. During the procedure, general behaviour, reaction to manipulation, and palpebral reflex were scored every five minutes.

All dogs were given 100% oxygen at a rate of 2 L/min by mask and Ringers’ Lactate Solution at a rate of 5 ml/kg/h (Ringer-Laktat-Lösung, Fresenius Kabi AG, Bad Homburg, Germany) during sedation. In case of insufficient sedation (awakening or movement) during the procedure, propofol (Propofol 1% MCT, Fresenius Kabi AG, Bad Homburg, Germany) was administered intravenously to effect in increments of 0.5 mg/kg. Propofol administration, heart rate, respiratory rate, pulse oximetry, and non-invasive blood pressure were recorded every five minutes. Heart rate and respiratory rate were evaluated by auscultation; pulse oximetry and non-invasive blood pressure were monitored by use of an anaesthesia monitor (A/S3, Datex Ohmeda, Anandic medical systems AG, Bern, Switzerland). In dogs undergoing skin biopsies, lidocaine 2% (Lidocain Streuli 2%, Streuli Pharma AG, Uznach, Switzerland; 1 mg/kg) was administered subcutaneously after onset of sedation. At the end of the procedure, the dogs were observed during recovery until they could stand up. Atipamezole (Antisedan, Orion Pharma, Finland; 0.02–0.1 mg/kg; equal volume as dexmedetomidine) was injected into the triceps muscle to antagonize the sedative effects of the alpha-2 agonist. The duration from the atipamezole injection to spontaneous sternal and, subsequently, spontaneous standing position was recorded. In dogs standing at the end of the procedure as well as in dogs waiting for a possible surgery, atipamezole was not administered.

A negative scoring system (NS) containing major and minor parameters was applied to evaluate the quality of sedation in each patient. Major parameters included scores for breathing (0–3; 0: normal breathing pattern, 3: apnea/need for intubation), signs of onset of sedation (0–2; 0: no changes in consciousness, 2: major disorientation, gagging, loud whining), need for propofol (0–3; 0: no need for propofol administration, 3: first dose of propofol required immediately/at positioning), cardiovascular changes (0–2; 0: no changes, 2: severe changes requiring drug administration), and behaviour during recovery (0–3; 0: uneventful, 3: major disorientation, panicking). Minor parameters included behavioural reactions during injection of the test drug, catheter placement, positioning on the table, atipamezole injection, an eventual need of a five-minute delay of procedure start as well as time spans to sternal and standing position (Table 3).

**Table 3:** Negative scoring system: Parameters were divided into major and minor scores

major		minor	
Respiration		Reaction to injection of the combination	
0	Normal breathing	0	No reaction
1	Nose stimulus necessary	1	Whining or slight moving
2	Several (nose) stimuli necessary	2	Loud whining +/- strong moving
3	Intubation necessary	Reaction to catheter placement	
Signs of onset of sedation		0	No reaction
0	No abnormalities	1	Looking
1	Muscle twitching, stiffness,	2	Whining or moving
	salivating	Delay of onset of sedation	
2	Major disorientation, gagging,	0	Start 20 min after injection
	loud whining	1	Five-minute delay
		Reaction to positioning on the table	
Need for propofol		0	No reaction
0	No need or 1st need after 40 min	1	Slight moving with the head
1	First need between 21–40 min	2	Moving with head and limbs
2	First need between 1–20 min	Reaction atipamezole injection	
3	First need at positioning	0	No reaction
Cardiovascular		1	Looking, twitching, whining
0	No severe changes	2	Loud whining or moving
1	MAP < 60	Time to sternal position after atipamezole injection	
1	HR < 50 (0-10 kg)	0	Within 0–10 min
1	HR < 40 (> 10 kg)	1	Within 11–20 min
2	Severe changes, drugs necessary	2	Within 21–30 min
Recovery		3	After 30 min
0	Uneventful	Time to standing position after atipamezole injection	
1	Little whining, slight disorientation	0	Within 0–30 min
2	Loud (continuing) whining	1	Within 31–60 min
3	Major disorientation, panicking	2	Within 61–90 min
		3	After 90 min

Major scores were summarized with a factor 1 whereas minor scores were summarized with a factor 0.5

The method applied in the present study was a modification of a model initially applied in human cancer patients by Berenbaum et al. [10]. Later, it has been adapted by Sveticic et al. to evaluate an analgesia protocol [13] before it was used to evaluate a sedation protocol in feline patients [12]. During phase 1 of the present evaluation, an initial complex consisting of eight combinations was empirically chosen. Every combination was tested in six dogs using the NS (Table 3). As a next step, every combination was ranked according to its NS. The combinations were then partitioned into a “promising” (P; low NS, low variability) or an “unsatisfactory” subgroup (U; higher NS, higher variability). A detailed description of the method is provided in the supplementary material.

For phase 2, the centroids of the two subgroups (P_c_ and U_c_ for the centroids of the “promising” and “unsatisfactory” subgroups, respectively) were determined and a new combination (N) was calculated using the formula N = P_c_ + α$*$(P_c_ – U_c_). Alpha was set at 1.3. The coefficient α defines the changes towards the final combination, away from the “unsatisfactory” combinations. Low α values induce small changes, requiring more steps to reach the end point. On the other hand, with large α values the optimum may be missed. According to findings from previous studies [13], we chose an α value of 1.3 for the current study.

Every new combination (N) was tested in six dogs and included in the next complex if ranked higher than the second to last combination of the previous complex. At the same time, the poorest combination of the previous complex was eliminated. However, when a new combination was ranked lower than the second to last, the combination was directly rejected and a new combination was calculated halfway between the centroid of the “promising” subgroup (P_c_) and N (i.e., 0.5(N + P_c_)). The same procedure was repeated for every new combination [10, 13].

Descriptive statistics were performed for all combinations, using a commercially available program (NCSS 2007). Results are reported as median values and interquartile ranges. One way ANOVA was used to compare the demographic data (age, weight) between groups and one way analysis of variance on ranks followed by Dunn’s method was applied to compare duration of sedation between groups. Significance was set at P < 0.05.

## Results

All 180 dogs completed the study and all dogs showed signs of sedation (physical and mental relaxation and tolerance to positioning) following the injection of the test drugs. In phase 1, 50 dogs were included, whereas in phase 2, a total of 30 dogs and in phase 3, 100 dogs were included in the study.

### Phases 1 (combinations A-H) and 2 (combinations G-L2)

**Fig. 1:**
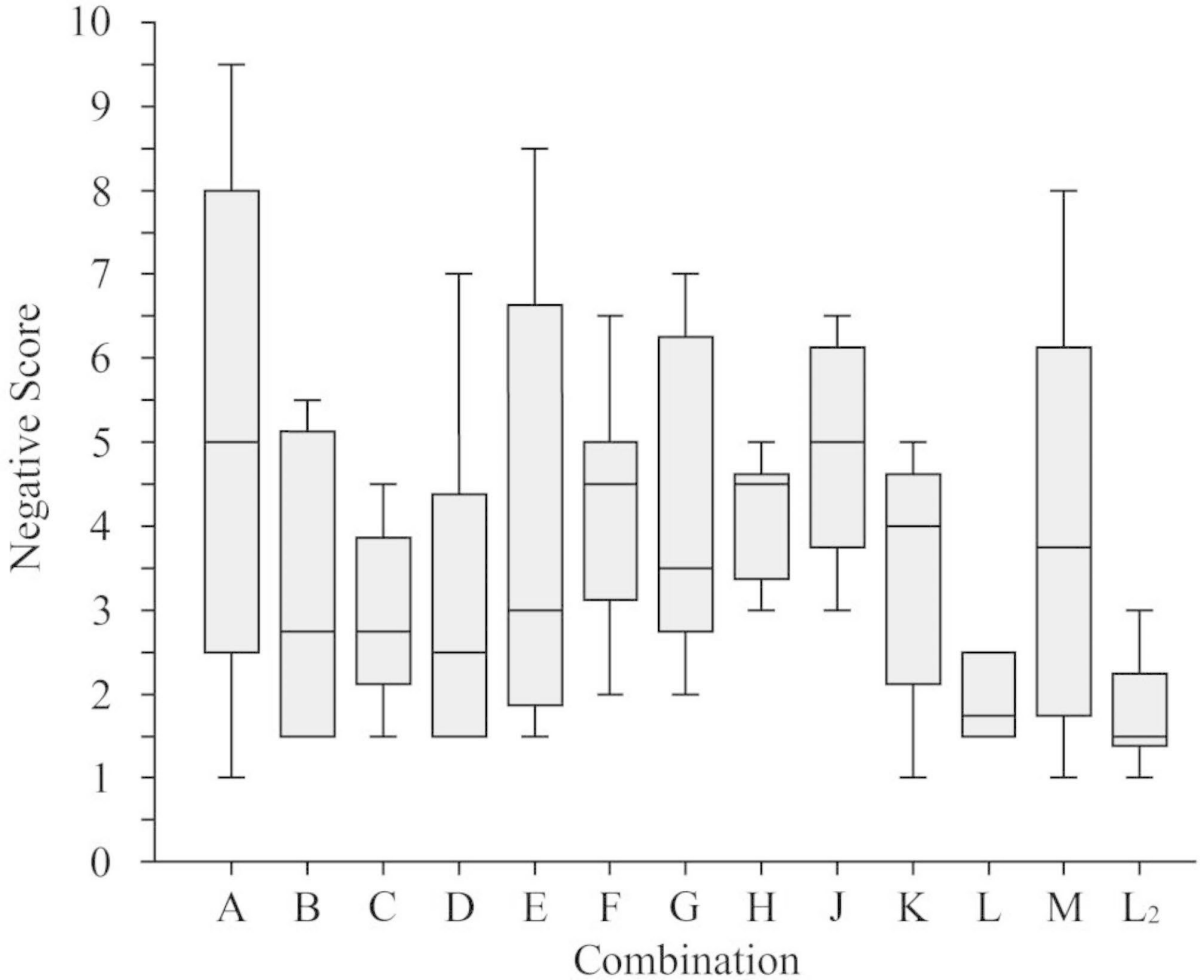
Median [interquartile range] negative score of six dogs per combination (eight dogs in C)

**Fig. 2:**
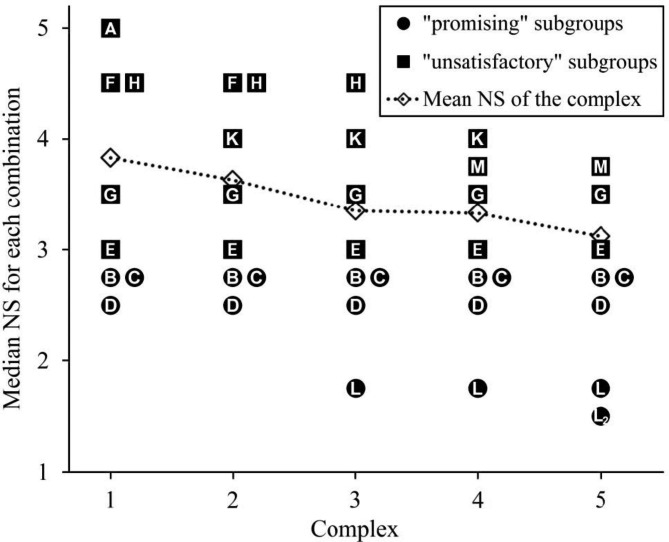
Median negative scores (NS) of all combinations allocated to complexes. Complexes were divided into “promising” and “unsatisfactory” subgroups. Means are calculated from raw data

In phase 1, the eight defined combinations were tested successfully in a blinded trial in 50 dogs (combination C was tested in eight instead of six dogs). Prior to the start of phase 2, the data were unblinded (Table 2). From the initial eight combinations, D was found to be the best combination (NS 2.5 [1.5-4.375]), followed by C (NS 2.75 [2.125–3.875]), B (NS 2.75 [1.5-5.125]), E (NS 3 [1.875–6.625]), G (NS 3.5 [2.75–6.25]), H (NS 4.5 [3.375–4.625]), F (NS 4.5 [3.125-5]), and A (NS 5 [2.5-8]) (Fig. 1). The first complex was divided into a “promising” (D, C, B) and an “unsatisfactory” subgroup (E, G, H, F, A) between the combinations B and E because of the higher median and larger interquartile range of the NS and because the only dog requiring intubation was in combination E (Fig. 2).

Phase 2 started with the calculation of the new combination J after determination of the centroids for both the “promising” and “unsatisfactory” subgroups (see Appendix). This mathematical procedure led to the exclusion of ketamine in combination J. Combination J (NS 5 [3.75–6.125]) was directly rejected after testing because of a higher NS (mainly due to insufficient sedation) than the second to last combination (F) in the first complex. Instead, a new combination K was calculated halfway between the centroid of the “promising” subgroup and J, in accordance with Berenbaum et al. [10]. The combination K (NS 4 [2.125–4.625]) was included into the second complex while A was eliminated. This second complex was partitioned into D, C, and B as the “promising” subgroup and E, G, K, H, and F as the “unsatisfactory” subgroup; also, a new combination L’ was calculated. However, the latter was rejected without testing because the dosage of dexmedetomidine was lower than in the previously rejected combination J. Therefore, a new combination (L) was calculated halfway between the centroid of the “promising” subgroup and L’. Combination L (NS 1.75 [1.5–2.5]) was used in the third complex, while F was eliminated. The newly calculated combination M’ was rejected without testing because of the lower dosages, but a new combination M was determined halfway between the centroid of the “promising” subgroup and M’. This combination M (NS 3.75 [1.75–6.125]) was included in the fourth complex, in which H was eliminated. The calculation of the next combination led back to combination M, and the halfway correction led back to combination L. This combination L2 was then tested in six dogs and was again rated as the best combination (NS 1.5 [1.375–2.25]). At this point, clinical testing was concluded because combination L was confirmed as the optimal combination.

No significant differences between the 13 groups could be detected when mean weight, mean age, and mean duration of sedation were compared (Table 4). Defensive reaction to injection was comparable in all groups. Combination L/L_2_ was found to be the optimal one. No correlation could be determined between NS and weight (*r* = 0.0788; P = 0.49) nor between NS and age (*r* = 0.0896; P = 0.43).

**Table 4:** Overview of all combinations used

Comb.	n	Sex, No. F/M	Mean age, yr (± SD)*	mean weight, kg (± SD)^§^	Type of procedure No. CT/Rx/Surg	mean duration of Sedation, min (± SD)#	1st propofol before No. 0/20/40/>40 min	Median HR Median RR Median SpO2 Median MAP
A	6	3/3	4 (± 2.66)	27.02 (± 20.01)	1/4/1	51.50 (± 14.28)	1/2/0/3	67.9 16.5 111.1 96.6
B	6	2/4	3.3 (± 2.77)	36.40 (± 29.57)	0/5/1	89.50 (± 46.09)	0/2/3/1	57 12.4 88.2 97.7
C	8	5/3	5.03 (± 3.12)	26.25 (± 14.68)	3/5/0	65.25 (± 15.22)	0/3/2/3	59.4 15.3 99.5 97.3
D	6	3/3	5.74 (± 2.47)	31.15 (16.93)	1/5/0	94.33 (± 16.83)	1/0/3/2	50.3 13.4 89.6 97.4
E	6	4/2	4.21 (± 2.66)	14.75 (± 8.41)	0/3/3	65.00 (± 34.19)	0/1/2/3	57.9 14.4 99.1 97.4
F	6	5/1	3.72 (± 2.66)	(31.83 (± 16.37)	1/5/0	82.17 (± 26.93)	1/1/2/2	58.6 15.7 93.6 97.7
G	6	1/5	6.40 (± 3.8)	40.81 (± 16.53)	2/3/1	72.17 (± 26.42)	0/0/1/5	53.3 11.0 112.1 96.6
H	6	5/1	2.38 (± 1.66)	26.62 (± 12.57)	1/2/3	51.83 (± 10.63)	0/0/1/5	58.6 12.9 108.6 96.8
J	6	3/3	3.66 (± 3.32)	22.95 (± 12.01)	0/4/2	69.50 (± 21.31)	1/1/3/1	37.6 13.4 88.7 97.6
K	6	4/2	4.42 (± 3.34)	30.00 (± 6.20)	0/5/1	77.83 (± 23.64)	0/1/1/4	49.4 12.0 95.9 97.2
L	6	4/2	4.41 (± 3.47)	35.22 (± 15.02)	1/4/1	80.17 (± 30.73)	0/0/3/3	46.4 11.8 87.8 97.6
M	6	3/3	5.50 (± 3.48)	27.95 (± 10.64)	2/4/0	80.83 (± 28.58)	2/1/1/2	45.5 14.5 101.0 96.9
L_2_	6	4/2	3.98 (± 1.56)	36.07 (± 10.34)	0/5/1	75.00 (± 23.77)	0/0/2/4	47.2 13.1 95.3 97.2
L_3_	100	47/53	4.37 (± 3.13)	32.21 (± 15.54)	75/3/25	71.98 (± 22.75)	0/1/27/29	57.8 15.5 96.7 86.9

*no significant difference between groups in terms of age (P = 0.617)

^§^no significant difference between groups in terms of weight (P = 0.35)

^#^no significant difference between groups in terms of duration of sedation (P = 0.05)

The empirically chosen combinations A-H were analyzed in the first complex. Combinations J-M were calculated during the stepwise optimization procedure. Ranges (minimum-maximum) are reported for age, weight, and duration of anaesthesia. The numbers of animals per category are shown for sex, type of procedure, and first propofol use. Types of procedures are categorized into computertomographic examinations (CT), radiographs (Rx), and small surgical procedures (Surg) such as skin biopsies, wound treatments or external fixation removals

Onset of sedation was fast in all groups: 72 dogs were in sternal position within 6 min and all dogs were in sternal position within 12 min after intramuscular injection. Venous catheter placement was possible in all dogs 10 min after injection as they became calm and tolerant to manipulation. None of the dogs required a second intramuscular injection.

In 66 dogs, depth of sedation was sufficient to start the procedure 20 min after injection. In eight dogs (1 in groups C, F and H; 2 in group D 3 in group J), a five minute delay was sufficient to allow for the procedure start, whereas in six dogs (1 in groups A, D, F and J; 2 in group M), propofol 0.5 mg/kg IV was required before starting the procedure. In one dog (group E), endotracheal intubation had to be performed (ten minutes after injection) and mechanical ventilation was necessary for 20 min until the respiratory efforts returned. In one dog (group G), seizure-like symptoms occurred after administration of the test drugs. The symptoms stopped immediately after an intravenous injection of diazepam (10 mg IV; Valium, Roche, Switzerland) was administered.

Continuous monitoring including oscillometric blood pressure, heart rate, electrocardiogram, respiratory rate, and oxygen saturation (SpO_2_) was started at the beginning of the procedure and measured in all dogs.

Mean heart rate of the whole population was 54 bpm (SD ± 15). No difference between the different groups could be detected (Table 4). In one dog of group K, ventricular tachycardia occurred. Treatment with lidocaine (Lidocaine 2%, Streuli, 2 mg/kg IV) led to immediate normalization of the heart rate.

During the first 20 min of the procedure, three dogs of group C, two dogs of groups A and B, and one dog of the groups E, F, J, K and M required propofol. After between 20 and 40 min into the procedure, propofol was administered to three dogs of groups B, D, J, and L, to two dogs of groups C, E, F, and L2, and to one dog of groups G, H, K and M (Table 4). No significant difference could be detected when the propofol doses were normalized to kilogram body weight. Of the twelve dogs tested with combination L, no dog showed any sign of apnoea, none of the dogs needed propofol administration within the first 20 min of the procedure, and only five dogs required propofol during the first 40 min.

Atipamezole was administered within five minutes after the end of the procedure. Only five dogs did not receive atipamezole: in three dogs, surgery was performed immediately afterwards, requiring general anaesthesia. One dog received an enema until awakening, and in one dog, ketamine carryover effects were suspected. Ten minutes after the atipamezole injection, 59 of 75 dogs were in sternal position or already standing; 20 min after injection, 68 of 75 dogs were in sternal position or standing; and 45 min after injection, all but one were at least in sternal position without external motivation. The latest dog to achieve spontaneous sternal position did so 82 min after atipamezole injection.

### Phase 3 (combination L_3_)

The optimal combination evaluated in phases 1 and 2 was found to be dexmedetomidine 0.005 mg/kg, ketamine 1 mg/kg, and butorphanol 0.3 mg/kg. This combination was then tested using the same NS in 100 cardiovascularly healthy dogs undergoing minor diagnostic or therapeutic procedures. The NS increased slightly with increasing procedure duration. The length of sedation was positively correlated with the recovery duration (R = 0.309; P = 0.002). No correlation could be detected between the NS and age (R = 0.113; P = 0.2619) and only a slight negative correlation could be seen between NS and weight (R = 0.211, P = 0.0348), as the NS slightly decreased with increasing body weight. To confirm an eventual negative correlation between body weight and NS, the NS would have required 250 dogs; to confirm an eventual correlation between age and NS, 600 dogs would have had to be included in the study.

Onset of sedation was fast as 73/100 dogs were in lateral recumbency within 10 min after intramuscular injection. Twenty minutes after injection, 88/100 dogs were in lateral recumbency, and depth of sedation was adequate to start the procedure. In 12 dogs, the start of the procedure had to be delayed by five minutes. In five of these dogs, a propofol bolus had to be administered before the procedure could be started.

Continuous monitoring was started at the beginning of the procedure. Oscillometric blood pressure, heart rate, respiratory rate, and oxygen saturation (SpO2) were measured in all dogs. In 80 dogs, mean arterial pressure (MAP) was higher than 60 mmHg at all points in time. In five dogs, MAP was lower than 60 mmHg and in 15 dogs, the MAP could not be measured due to problems related to equipment failure.

In those dogs weighing ≤ 10 kg (8 animals), the mean HR decreased from 126 bpm prior to sedation to 65 bpm at the beginning of the procedure. During the procedure, the mean HR was 60 bpm. After recovery, the mean HR returned to 111 bpm. In dogs weighing 10–31 kg (36 animals), the mean HR decreased from 97 bpm prior to sedation to 59 bpm at the beginning of the procedure. In this group, the mean HR was 55 bpm during the procedure. In four dogs, the mean HR was < 40 bpm during the whole procedure; however, after recovery, the mean HR returned to 83 bpm. In dogs weighing > 31 kg (55 animals), the mean HR decreased from 98 bpm prior to sedation to 60 bpm at the beginning of the procedure. During the procedure, the mean HR was 43 bpm. In 73 dogs weighing ≥ 10.1 kg, the HR remained at levels > 40 beats per minute. In 24 animals, severe bradycardia (HR < 40 bpm) occurred (five dogs < 10 kg and 19 dogs > 10 kg). Nevertheless, due to adequate oxygen saturation (SpO2 > 95%) and a mean blood pressure within physiological range, no further measures were taken. In one dog, premature ventricular complexes were detected and in two dogs, a second-degree AV block could be observed.

Oxygen saturation was measured in 89/100 dogs. In 42/89 dogs, the value was < 95% at the beginning of the procedure. Oxygen saturation reached a value of ≥ 95% in 87/89 dogs fifteen minutes after drug injection. In the remaining two dogs, at the same point in time, oxygen saturation was at 91% and 92%, respectively, but ≥ 95% at 30 min.

Propofol (0.5 mg/kg) was administered intravenously as soon as movements or a reaction to manipulation were observed. In 43 dogs, no propofol had to be administered at any point in time. In three dogs, propofol was injected ≥ 40 min after start of the procedure. In 18 dogs, it was administered between after 21–40 min into the procedure. In 30 dogs, propofol was administered within the first 20 min of the procedure. In six dogs, propofol had to be injected before the procedure could be started.

All dogs recovered from sedation. In 77 cases, recovery was uneventful. In 20 animals, loud (continuous) whining for some minutes was recorded. One dog showed major disorientation, which also disappeared within minutes. Atipamezole was injected intramuscularly to 79 dogs. In animals already standing at the end of the procedure, atipamezole was omitted (21 animals).

## Discussion

According to the optimization method applied in this trial, for cardiovascularly healthy dogs undergoing diagnostic or minor surgical procedures, the combination of dexmedetomidine 0.005 mg/kg, ketamine 1 mg/kg, and butorphanol 0.3 mg/kg offered the best quality of sedation with the least side effects and a minimal need for additional anaesthetics. The combination was then evaluated in a larger clinical trial. The optimization method used in the present study was easily compatible with the clinical needs in a veterinary teaching hospital setting.

Tomizawa et al. [28] compared medetomidine, butorphanol, and ketamine to medetomidine and ketamine alone and observed a reduced induction time and an improved analgesic effect when butorphanol was added. Barletta et al. [32] found no difference in intubation, anaesthesia, pain, and recovery scores when comparing dexmedetomidine and ketamine in combination with various opioids in a single IM injection for castration in dogs. On the other hand, various combinations of medetomidine (dose range 0.02–0.05 µg/kg) and ketamine (dose range 2.5–10 mg/kg)[19–21, 33] as well as medetomidine (dose range 5–22 µg/kg) and butorphanol (dose range 0.1–0.22 mg/kg) have been tested [20, 33–37].

In the present study, the ratio of dexmedetomidine to ketamine had a major influence on bradycardia and ketamine side effects such as muscle twitching, stiffness, salivation, disorientation, or whining. Without ketamine (group J), all but one dogs showed severe bradycardia at least once during the procedure (as defined in Table 2). When the ratio of ketamine (mg/kg) to dexmedetomidine (µg/kg) was greater than 1 (combinations A and F), only one dog experienced severe bradycardia starting 40 min after injection. However, a tendency towards more ketamine side effects could be detected at the onset of sedation in these combinations.

Body weight was used to calculate dosages of dexmedetomidine in this study, although Vähä-Vahe [38] reported a lower satisfaction with the overall suitability of medetomidine in small-breed dogs compared to larger breeds in a clinical study including 1736 dogs. As a consequence, several authors have calculated dexmedetomidine dosages according to body surface area rather than to body weight [39–42], whereas others have continued to calculate dosages strictly according to body weight [12, 32, 43–45]. In the present study, no influence of weight on the NS was detected in the phases 1 and 2 (the correlation between the NS and weight was < 0.1) while in phase 3, a slightly negative correlation could be determined, suggesting that the calculation of dexmedetomidine dosages based on body surface area would be preferable.

The aim of this study was to improve the quality of sedation by use of an objective method. Therefore, a negative scoring system was purposefully developed (Table 2). There are several scoring systems to judge various parameters of sedation [46–48], but none of these systems have been tested in clinical trials but in none of them behavioural and physiological parameters were monitored during the entire procedure. Various parameters were considered in the NS, but not all parameters were equally important, leading to the implementation of a factor used to differentiate between major and minor parameters. Major parameters had a more enduring and profound effect on the animal’s well-being, whereas minor parameters concerned procedural matters such as handling and timing. Variability among observers using this NS is expected to be low. However, during phase 2 and 3 of the study, the person observing the dog was aware of the combination administered. This constitutes a limitation of this study. In contrast, in the study of Sveticic [49], the human patients communicating their level of pain as well as all directly involved physicians remained unaware of the combination administered.

Originally recommended by Box [50], a value of 1.3 for the factor α had been applied in various medical optimization studies [11, 49, 51] and was used in the present study as well. However, after concluding the clinical part of the study, we have come to suppose that a value between 0.5 and 0.8 might have been more appropriate for our setting, seeing that none of the newly calculated dosages were satisfactory and thus had to be replaced by combinations halfway between the centroid of the “promising” subgroup and the calculated combinations. A smaller value for α might be beneficial if the initially chosen dosages are rather high and if the differences between the centroids of the “promising” and “unsatisfactory” subgroups are large.

An important limitation to our study is the wide array of procedures included in the study. Indeed, the duration and nociceptive stimulation between the different procedures may bias the requirements for additional pain therapy during the procedure. A surgical biopsy is longer and more invasive with a higher nociceptive stimulus than diagnostic procedures such as radiographs or a joint palpation under sedation. The mean duration of the procedures was 43 min; a slight correlation between the duration of sedation and the duration of recovery could be detected. Due to the large variability of procedures performed in the animals, it was impossible to standardize the length of the procedures and the intensity of stimulation. Depending on the reason for the diagnostic procedure, even radiographs required highly variable manipulations. The point in time of the first propofol injection correlated slightly with the intensity as well as with the frequency of stimulation. A decreased sensitivity to the test drugs was suspected in severely stressed dogs, but cannot be avoided in a clinical population [42].

## Conclusions

The application of the stepwise optimization method led to the definition of a reliable and safe combination of drugs to be used for sedations suitable for various diagnostic and therapeutic examinations. Our data suggest that the optimal combination offers a good quality of sedation with minimal side effects and a minimal need for additional anaesthetics. Even though the combination was then tested in 100 healthy dogs undergoing minor diagnostic and therapeutic procedures, it should now be further evaluated in a larger clinical trial to verify its clinical utility and to evaluate the incidence of serious side effects.

### Electronic supplementary material

Below is the link to the electronic supplementary material.

Additional file 1. Detailed declaration of the drug calculations by use of the direct search model.

## Data Availability

The datasets used and/or analyzed during the current study are available from the corresponding author on reasonable request.

## Declarations

### Prior publication

The data have not been published previously.
