# Supplementary material for: Determination of a safe sedative combination of dexmedetomidine, ketamine and butorphanol for minor procedures in dogs by use of a stepwise optimization method
Source: Acta Vet Scand. 2023 Sep 22;65:41. doi: 10.1186/s13028-023-00697-8 (PMC10515426; doi:10.1186/s13028-023-00697-8)
Supplement: Supplementary file 1 — Additional file 1. Detailed declaration of the drug calculations by use of the direct search model. [file 13028_2023_697_MOESM1_ESM.docx]

**Additional file 1: Detailed declaration of the drug calculations by use of the direct search model**

The number of combinations (m=8) forming each complex and the number of dogs tested per combination (n=6) were chosen according to Sveticic et al [49]. In order to compare the combinations, a negative score (NS) was established (Table 3). The median of the NS of each group was taken to rank the combinations. The interquartile range was considered in case of equal medians of the NS of two combinations.

After completing phase 1, which included scoring 50 dogs (combination C was tested in eight instead of six dogs), the blinding code was opened (Table 2). From the initial eight combinations, D was the best combination (2.5 [1.5-4.375]), followed by C (2.75 [2.125-3.875]), B (2.75 [1.5-5.125]), E (3 [1.875-6.625]), G (3.5 [2.75-6.25]), H (4.5 [3.375-4.625]), F (4.5 [3.125-5]), and A (5 [2.5-8]) (Figure 2).

NS_D_<NS_C_<NS_B_<NS_E_<NS_G_<NS_H_<NS_F_<NS_A_

As suggested by Sveticic et al [[50](#_ENREF_50)], the complex was not partitioned simply by cutting it in half but according to differences in medians, interquartile ranges, and important events occurring while testing the combinations. The first complex was dichotomized into a “promising” (D, C, B) and an “unsatisfactory” (E, G, H, F, A) subgroup. The cut point of the subgroups was made between the combinations B and E because of the higher median and larger interquartile range of the NS and because the only dog requiring intubation was in combination E (Figure 3).

As previously described [[32](#_ENREF_32), [50](#_ENREF_50)], the centroids for each subgroup (P_c_ and U_c_ for the centroids of the “promising” and “unsatisfactory” subgroups, respectively) were calculated by computing the mean of both variables dexmedetomidine (d) and ketamine (k). For instance, P_c_^d^ was (8+6+4)/3 = 6, P_c_^k^ was (1+2+3)/3 = 2, U_c_^d^ was (10+8+10+4+2)/5 = 6.8 and so forth. The new combination (N) was calculated as previously described [[32](#_ENREF_32), [50](#_ENREF_50)].

N = P_c_ + α$*$(P_c_ – U_c_)

When α > 0. The value for α (1.3) has been used in various medical studies [[32](#_ENREF_32), [50-52](#_ENREF_50)] recommended this in their optimization method.

Full mathematics for the calculation of the new combination J from the first complex:

P_c_ = $\left\{ \binom{8}{1}+\binom{6}{2}+\binom{4}{3} \right\} :3=\binom{18}{6} :3= \binom{6}{2}$

U_c_ = $\left\{ \binom{10}{5}+\binom{8}{3}+\binom{10}{2}+\binom{4}{6}+\binom{2}{4} \right\} :5=\binom{34}{20} :5=\binom{6.8}{4}$

N = P_c_ + α$*$(P_c_ – U_c_) = $\binom{6}{2}+$1.3$*\left\{ \binom{6}{2}-\binom{6.8}{4} \right\}$ = $\binom{6}{2}$ + 1.3$*\binom{-0.8}{-2}$ = $\binom{6}{2}$ – 1.3$*\binom{0.8}{2}$ = $\binom{6}{2}$ – $\binom{1.04}{2.6}$ = $\binom{4.96}{-0.6}$which is rounded to $\binom{5}{0}$ = J

This mathematical procedure led to the exclusion of ketamine in combination J. As described by Berenbaum [[32](#_ENREF_32)], this combination J (5 [3.75-6.125]) was directly rejected after testing because of a higher NS (mainly due to insufficient sedation) than the second to last combination (F) in the first complex.

NS_D_<NS_C_<NS_B_<NS_E_<NS_G_<NS_H_<NS_F_<NS_J_

Berenbaum [[32](#_ENREF_32)] presumed in this case that the search path has overshot the optimum and a new combination should be calculated halfway back between the new combination (J) and the centroid of the “promising” subgroup (P_c_) (i.e. 0.5(N+P_c_))

0.5$*$(J+P_c_) = 0.5 $*\left\{ \binom{5}{0}+\binom{6}{2} \right\}$ = 0.5 $*\binom{11}{2}$ = $\binom{5.5}{1}$ ≈ $\binom{6}{1}$ = K

This combination K (4 [2.125-4.625]) was included into the second complex after testing while A was eliminated.

The combinations were ranked again:

NS_D_<NS_C_<NS_B_<NS_E_<NS_G_<NS_K_<NS_H_<NS_F_

The partitioning was performed between B and E again and a new combination was calculated. Because this combination (L’) was $\binom{4}{0}$ and the combination $\binom{5}{0}$ = J had already been tested and rejected (due to insufficient sedation), testing of this new combination was abandoned and a new combination was calculated halfway between the centroid of the “promising” subgroup and L’.

0.5$*$(L’+P_c_) = 0.5 $*\left\{ \binom{4}{0}+\binom{6}{2} \right\}$ = 0.5 $*\binom{10}{2}$ = $\binom{5}{1}$ = L

Combination L (1.75 [1.5-2.5]) was accepted and combination F was replaced by combination L, which was ranked as the best combination.

NS_L_<NS_D_<NS_C_<NS_B_<NS_E_<NS_G_<NS_K_<NS_H_

Again, the partitioning was performed between B and E, creating two subgroups of four combinations each. The mathematical procedure resulted in a new combination M’ = $\binom{2}{0}$ which was again not tested but replaced by a combination halfway between M’ and the new centroid P_c_ (including L).

0.5$*$(M’+P_c_) = 0.5 $*\left\{ \binom{2}{0}+\binom{5.75}{1.75} \right\}$ = 0.5 $*$ $\binom{7.75}{1.75}$ = $\binom{3.875}{0.875}$ ≈ $\binom{4}{1}$ = M

This combination was accepted and combination H was replaced by M (3.75 [1.75-6.125]) in the third complex, but M was ranked in the “unsatisfactory” subgroup as the cut off for partitioning was again between B and E.

NS_L_<NS_D_<NS_C_<NS_B_<NS_E_<NS_G_<NS_M_<NS_K_

The following calculation again resulted in combination M=$\binom{4}{1}$=N’, which was not retested but another step backwards was made.

0.5$*$(N’+P_c_) = 0.5 $*$ $\left\{ \binom{4}{1}+\binom{5.75}{1.75} \right\}$ = 0.5 $*\binom{9.75}{2.75}$ = $\binom{4.875}{1.375}$ ≈ $\binom{5}{1}$ = L_2_

This combination L_2_ was then re-tested in six dogs and was again rated as the best combination (1.5 [1.375-2.25]). At this point, clinical testing was concluded because combination L was confirmed as the optimal combination.
